# Prolonged quiescence delays somatic stem cell‐like divisions in *Caenorhabditis elegans* and is controlled by insulin signaling

**DOI:** 10.1111/acel.13085

**Published:** 2019-12-18

**Authors:** María Olmedo, Alejandro Mata‐Cabana, María Jesús Rodríguez‐Palero, Sabas García‐Sánchez, Antonio Fernández‐Yañez, Martha Merrow, Marta Artal‐Sanz

**Affiliations:** ^1^ Departamento de Genética Facultad de Biología Universidad de Sevilla Seville Spain; ^2^ Andalusian Center for Developmental Biology Consejo Superior de Investigaciones Científicas/Junta de Andalucía/Universidad Pablo de Olavide Seville Spain; ^3^ Department of Molecular Biology and Biochemical Engineering Universidad Pablo de Olavide Seville Spain; ^4^ Institute of Medical Psychology Faculty of Medicine LMU Munich Munich Germany

**Keywords:** arrest, *C. elegans*, development, insulin signaling, proliferation, quiescence

## Abstract

Cells can enter quiescence in adverse conditions and resume proliferation when the environment becomes favorable. Prolonged quiescence comes with a cost, reducing the subsequent speed and potential to return to proliferation. Here, we show that a similar process happens during *Caenorhabditis elegans* development, providing an in vivo model to study proliferative capacity after quiescence. Hatching under starvation provokes the arrest of blast cell divisions that normally take place during the first larval stage (L1). We have used a novel method to precisely quantify each stage of postembryonic development to analyze the consequences of prolonged L1 quiescence. We report that prolonged L1 quiescence delays the reactivation of blast cell divisions in *C. elegans*, leading to a delay in the initiation of postembryonic development. The transcription factor DAF‐16/FOXO is necessary for rapid recovery after extended arrest, and this effect is independent from its role as a suppressor of cell proliferation. Instead, the activation of DAF‐16 by decreased insulin signaling reduces the rate of L1 aging, increasing proliferative potential. We also show that yolk provisioning affects the proliferative potential after L1 arrest modulating the rate of L1 aging, providing a possible mechanistic link between insulin signaling and the maintenance of proliferative potential. Furthermore, variable yolk provisioning in embryos is one of the sources of interindividual variability in recovery after quiescence of genetically identical animals. Our results support the relevance of L1 arrest as an in vivo model to study stem cell‐like aging and the mechanisms for maintenance of proliferation potential after quiescence.

## Graphical abstract

Working model of the regulation of L1 aging and its effect on the initiation of postembryonic development. Reduced insulin signaling increases vitellogenin provisioning in embryos. After hatching, these L1 larvae age at a slower rate, allowing fast initiation of larval development. Increased L1 aging leads to a delay in the reactivation of proliferation, represented by the delayed initiation of seam cell divisions. Development proceeds at similar speed, independently of the rate of L1 aging.

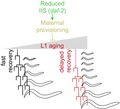

## 1 INTRODUCTION

In natural conditions, organisms are often subjected to changes in nutrient availability that modulates growth and proliferation. In the absence of growth‐sustaining factors, cells can enter quiescence, a reversible, nonproliferative state. Interestingly, cellular quiescence also occurs in normal development, with many cells spending most of their lifetime in this state. Under certain conditions, quiescent cells re‐enter the cell cycle and resume proliferation (McCulloch & Till, 2005; Siminovitch, McCulloch, & Till, 1963). Despite the lack of proliferation, quiescence is not a homogeneous state. Cells move progressively into a deeper level of quiescence over time (Kwon et al., 2017). As a consequence, the duration of quiescence affects cell viability and proliferation potential. Understanding the molecular processes that regulate the maintenance of proliferation potential and the mechanisms mediating reactivation of proliferation after quiescence will contribute to comprehension of stem cell aging. Furthermore, failure of the programs that negatively regulate cell division upon withdrawal of growth signals would lead to uncontrolled proliferation, a hallmark of cancer cells (reviewed in (Hanahan & Weinberg, 2011)).

In the nematode *C. elegans*, newly hatched larvae enter a quiescent state when subjected to food deprivation. These L1 larvae (first larval stage) have 558 nuclei, 53 of which are in somatic blast cells that divide further to reach the 959 somatic cells of the adult (Sulston & Horvitz, 1977). In the presence of food, blast cells divide over four stages of postembryonic development (L1‐L4), and somatic cell divisions end with the transition to adulthood. When embryos hatch in the absence of food, they arrest at the L1 stage. This arrest involves quiescence of the blast cells that would normally divide during L1. The penetrance of the arrest depends on the cyclin‐dependent kinase inhibitor CKI‐1/CIP/KIP/p27 that holds the cell cycle at G1 (Hong, Roy, & Ambros, 1998). The activation of CKI‐1 in the absence of food is in part mediated by DAF‐16/FOXO, a transcription factor activated in conditions of low insulin signaling. A fraction of *daf‐16* mutant starved L1 larvae partially initiate divisions of postembryonic development (Baugh & Sternberg, 2006), although this only occurs in the presence of small amounts of ethanol (Fukuyama, Kontani, Katada, & Rougvie, 2015).

Arrested L1 larvae can survive several weeks without food and show increased resistance to stress (Baugh, 2013). Following feeding after prolonged L1 starvation, animals take longer to reach adulthood and show increased variability in the time it takes to reach this developmental stage (Jobson et al., 2015; Lee, Hendrix, Kim, Yoshimoto, & You, 2012). During L1 arrest, larvae undergo a process of aging, manifested by the accumulation of protein aggregates, increased reactive oxygen species (ROS) accumulation, and mitochondrial fragmentation. The addition of food to arrested L1 leads to the reversion of these aging phenotypes and the initiation of larval development. However, extended L1 arrest reduces the potential for recovery (Roux, Langhans, Huynh, & Kenyon, 2016). Sleep during L1 arrest counteracts aging phenotypes and increases survival rates (Wu, Masurat, Preis, & Bringmann, 2018). Thus, the nematode offers an exceptional tractable model system to study the mechanisms that impact cell arrest and proliferation in a multicellular organism.

Here, we show that prolonged L1 arrest delays the reactivation of the cell divisions that mark the initiation of the postembryonic developmental program. Once the program is initiated, our novel and highly quantitative assay shows that developmental timing is not affected. This contrasts with previous interpretations concerning the delay in reaching adulthood observed in animals after prolonged starvation. The specific effect of prolonged quiescence in recovery is maintained over the lifespan of arrested L1. In our effort to understand the signaling pathways controlling quiescence, we investigated the role of insulin signaling in recovering from L1 arrest. Low insulin signaling in the insulin receptor mutant *daf‐2* leads to faster recovery and reduced aging, suggesting a shallower level of quiescence. The transcription factor DAF‐16 is necessary for the effect of the *daf‐2* mutation in recovery. DAF‐16 function in the process is not related to a possible role as a suppressor of proliferation but to its role activating stress responses. Finally, we have discovered that maternal provisioning, that is also regulated by insulin signaling, reduces aging and improves proliferation after prolonged arrest. With this new approach to study re‐initiation of cell proliferation after arrest, we have added an in vivo model that will contribute to tackle the mechanisms controlling maintenance of proliferation potential after arrest in multicellular organisms.

## 2 RESULTS

### 2.1 Time to recover from L1 arrest increases with prolonged starvation

**Figure 1:**
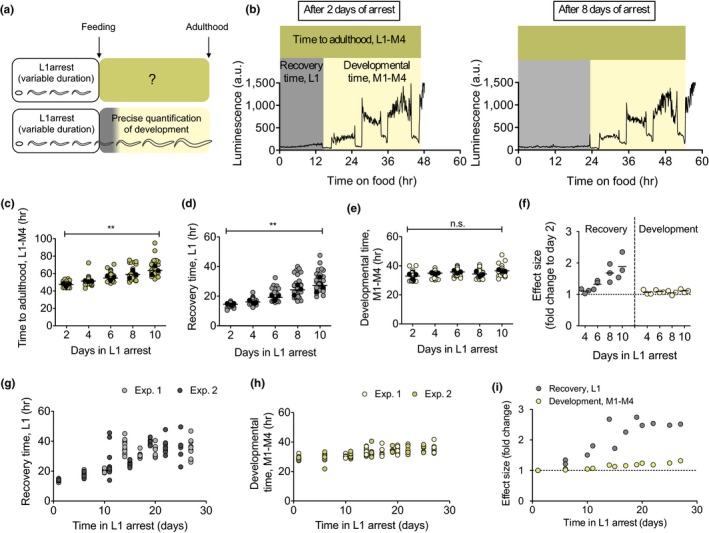
Prolonged quiescence delays recovery time. (a) Past and current experimental designs to study the consequences of prolonged L1 arrest. (b) Representative plots of the duration of development for animals arrested as L1 for 2 days (left) and 8 days (right). Recovery time (L1) is defined as the time between the addition of food to starved L1 animals and the initiation of the first molt. Developmental timing is defined as the period between the beginning of the first molt and the end of the last molt, or initiation of adulthood (M1‐M4). Total time to reach adulthood is the time between the addition of food and the end of the last molt (L1‐M4). (c–e) Total time to adulthood (c), recovery time (d), and developmental time (e) for larvae arrested for 2–10 days. Average values per experiment are indicated with a black dot, and values from single animals are indicated with a colored dot. We performed one‐way ANOVA on the averages of 3 biological replicates (** *p* < .01). (f) Effect size of prolonged starvation in recovery and development. The data show the ratio of the average duration relative to day 2, for the three independent experiments shown in 1c‐e. (g–h) Recovery (g) and developmental (h) time of L1 arrested up to 27 days. The plots show data from two independent replicates. (i) Effect size of prolonged starvation in recovery and development

When L1 larvae are arrested for prolonged periods of time, animals take longer to reach adulthood once they are fed (Jobson et al., 2015; Lee et al., 2012). However, the duration of each of the four larval stages after extended starvation remains unexplored, hindering the analysis of the effects of starvation on the reactivation of the larval developmental program. To investigate in detail the timing of development after starvation, we used a quantitative and novel assay based on the use of a luciferase reporter strain (Olmedo, Geibel, Artal‐Sanz, & Merrow, 2015). The enzyme luciferase, which is constitutively expressed in this strain, catalyzes the oxidation of the substrate luciferin, provided with the food. During the transitions between the larval stages, *C. elegans* ceases feeding and does not incorporate the substrate, leading to a rapid reduction in the luminescence signal. The profile of light emission can be used to precisely determine the timing of larval stages and molts. We measured the duration of all larval stages after different periods in L1 arrest (Figure 1a,b). As previously observed, the entry into adulthood was increasingly delayed with longer times in arrest (Figure 1c). When we analyzed the duration of each stage of development, we found that, of all larval and molt stages, only L1 is affected by the duration of the arrest (Figure S1). Delays in reaching adulthood resulted from an extended recovery time, defined as the time from the exposure to food until the entry into the first molt (L1; Figure 1d). Prolonged starvation also resulted in increased variance of recovery time (*F* test *p*‐value < .0001, when comparing day 2 and day 8) (Figure 1d). Starvation time had no effect on the amount of time between the first and the last molt (M1‐M4) (Figure 1e). The differential effect of prolonged starvation on recovery time and development becomes evident when comparing the effect size for both processes (Figure 1f). Furthermore, we extended the time of starvation up to 27 days to analyze whether the effect on recovery and development was the same for any duration of the arrest. Despite a modest increase in the duration of development after very prolonged arrest, the effect is not comparable to the lengthened recovery time (Figure 1g,h). The maximum delay in development is about 30%, while the extension of the recovery period reaches 250% (Figure 1i). In summary, developmental timing is largely resilient to extended arrest, whereas recovery time increases with the duration of the arrest.

### 2.2 Recovery time reflects proliferative potential after quiescence

**Figure 2:**
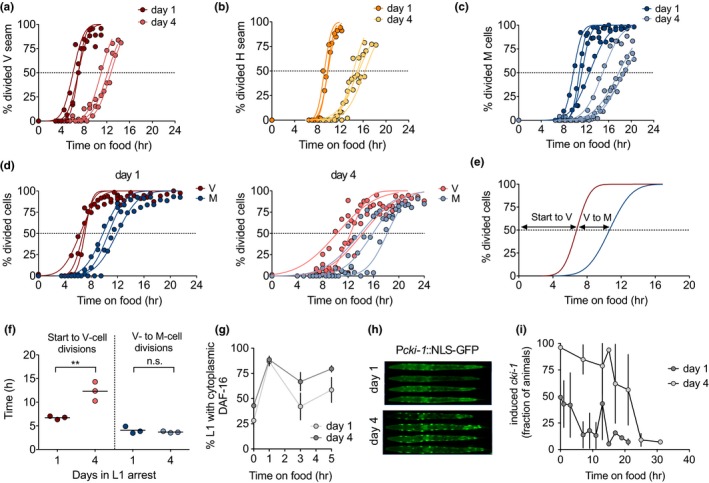
Recovery time reflects reactivation of the developmental program. (a–c) Timing of seam cells (a,b) and M cell (c) division upon addition of food to L1 larvae arrested for one day or for four days. The plots show data from 3–4 biological replicates, and values represent the percentage of animals showing division. Curves represent the fit assuming a cumulative Gaussian distribution. The dashed lines indicate the value of 50% of animals with divided cells. (d) Timing of division of V seam cells and M cells upon addition of food to L1 larvae arrested for one day or for four days. We analyzed a double reporter to assay both divisions in the same animals, performing three biological replicates. (e) Representative plot showing the calculated intervals between cell divisions. (f) Prolonged arrest delays the division of V seam cells but not the timing between V‐ and M‐cell divisions. (g) Percentage of animals with cytoplasmic localization of DAF‐16 in the first five hours upon the addition of food after one or four day of L1 arrest. The plot shows the mean (±*SD*) of four biological replicates. (h) Representative images of *cki‐1* activation after one and four days of L1 arrest. (i) Activation of *cki‐1* during recovery after one or four days of arrest. The plot shows the average (±*SD*) of three independent replicates

In order to understand the nature of the extended L1 stage, we fitted the recovery times after different starvation durations using linear regression. The stage L1, by definition, starts with hatching of the embryos and ends with the transition to L2. However, this definition does not consider the time needed to launch the postembryonic developmental program. As a consequence, L1 appears longer than the rest of larval stages. The relative molt/larva duration is markedly smaller for M1/L1, compared to all the other larval stages (Olmedo et al., 2015). From the linear regression, we calculated the time (*y* value) needed to reach the first molt when time in arrest (*x*) equals 0. This provides an estimate of the *bonafide* duration of L1 for nonstarved larvae of *ca.* 10 hr (Figure S2 a). With the *bonafide* L1 duration determined from our data, the M1/L1 ratio reached the same value as all the other larval stages (Figure S2 b). Our hypothesis is that prolonged L1 arrest does not affect the duration of the *bonafide* L1, but it determines the delay between re‐introduction of food and reactivation of postembryonic cell divisions. Alternatively, after extended arrest, reactivation of cell divisions could take place upon food addition without a delay and the complete L1 stage be prolonged (Figure S2 c). To differentiate between these two possibilities, we analyzed the relative timing of specific events during L1, namely, the divisions of seam and M cells. Seam cells undergo a single round of asymmetric self‐renewing division at the beginning of each larval stage (L1‐L4). The first of these divisions takes place about five hours after hatching. M blast cells present in the L1 larvae give rise to the complete mesodermal cell lineage. The first division of the M cell takes place midway through this larval stage (Sulston & Horvitz, 1977). We used fluorescent reporters to monitor the timing of seam cells (both V and H lineages) and M‐cell division after one and four days of arrest. Specifically, we used *Pscm::gfp* reporters to follow the division of seam cells and a *Phlh‐8::gfp* reporter to monitor M cells divisions*.* After a single day of arrest, seam cells of the V1‐4 and H lineages start to divide after five and eight hours, respectively, following exposure to food (Figure 2a–b). M cells divide after 9 hr (Figure 2c). After four days of arrest, the divisions were delayed on average 5.75 hr in the case of seam cells and 6.43 hr in the case of the M cells. These results support the first scenario, in which relative timing of divisions is similar whether animals are arrested only briefly or for extended periods. To compare the time of blast cell divisions within the same animals, we made a double‐seam and M‐cell reporter and monitored V‐ and M‐cell divisions upon feeding of larvae arrested for one and four days (Figure 2d). We calculated the time needed for 50% of animals to achieve these cell divisions in the two conditions (Figure 2e). While the time between the addition of food and V cell divisions is almost doubled in L1s arrested for four days, the time between V‐ and M‐cell divisions remains constant (Figure 2f). We concluded that reactivation of blast cells is delayed after prolonged quiescence in *C. elegans*. This result supports our hypothesis that the *bonafide* L1 is constant over time in arrest, as are the rest of larval stages. As a consequence, recovery time can be used to quantify proliferative potential after quiescence. These findings support L1 arrest as an in vivo model to study proliferation reactivation potential after cell quiescence.

We asked whether delayed cell division after prolonged arrest resulted from a failure to release the repression exerted by DAF‐16. The number of animals with cytoplasmic localization increases rapidly in response to food independently of the duration of the arrest (Figure 2g), suggesting that exposure to food is not sufficient to resume the larval developmental program. The cyclin kinase inhibitor CKI‐1 mediates developmentally timed cell cycle arrest, as well as the arrest meditated by starvation, partially under the control of DAF‐16 (Baugh & Sternberg, 2006). We visualized *cki‐1* expression in the seam cells of arrested L1, which was higher after prolonged arrest (Figure 2h). When we fed L1 larvae after arrest for either one or four days, those subjected to shorter arrest reached basal *cki‐1* levels after less than 10 hr, while L1 arrested for four days needed more than 20 hr to reach similar levels (Figure 2i). These results imply that the rapid cytoplasmic relocation of DAF‐16 upon feeding after four days of arrest is not sufficient to produce an immediate reduction of *cki‐1* expression. Other processes must be hindering CKI‐1 reduction in response to food and delaying proliferation after prolonged L1 quiescence.

### 2.3 Insulin signaling modulates L1 aging and recovery

**Figure 3:**
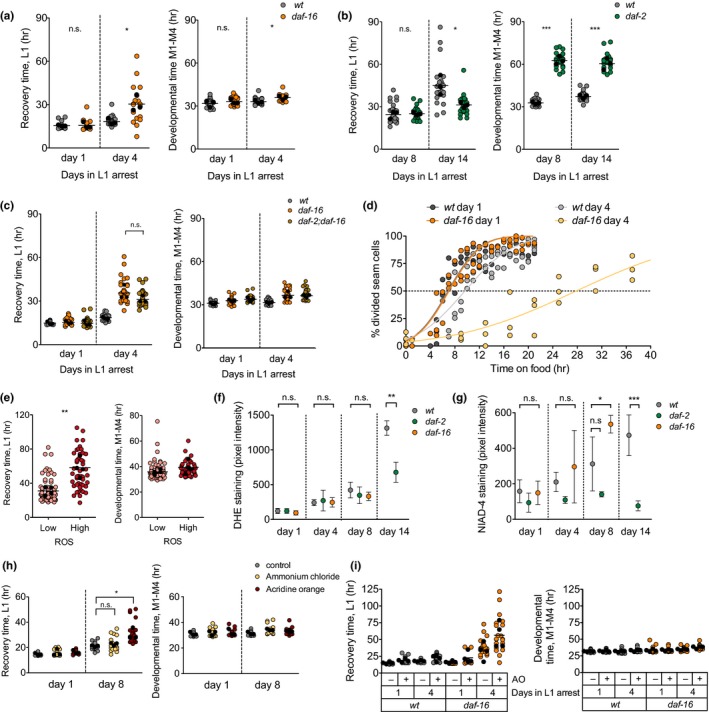
Low insulin signaling during quiescence ameliorates proliferative potential and attenuates L1 aging. (a) Recovery and developmental time for the wild‐type strain and the *daf‐16(mu86)* mutant after one and four days of arrest. Average values per experiment are indicated with a black dot, and values from single animals are indicated with a colored dot. We performed *t* test on the averages of 3 biological replicates (* *p* < .05). (b) Recovery and developmental timing for the wild‐type strain and the *daf‐2(e1370)* mutant after 8 and 14 days of arrest. We performed *t* test on the averages of 3 biological replicates (* *p* < .05, *** *p* < .001). (c) Recovery and developmental timing for the wild‐type strain, the *daf‐16(mu86)* mutant and the double‐mutant *daf‐2(e1370);daf‐16(mu86)* mutant after 1 and 4 days of arrest. (d) Seam cell division in wild‐type and *daf‐16* larvae during recovery after one or four days of arrest. Values represent the percentage of animals showing division in three biological replicates. (e) Recovery and developmental timing for animals categorized as having low or high DHE staining after 8 days of arrest. We performed one‐way ANOVA on the averages of 4 biological replicates (** *p* < .01). (f) Quantification of ROS accumulation in the wild‐type strain, *daf‐2 (e1370)*, and *daf‐16(mu86)*. Plots show mean (±*SD*) of 3–4 biological replicates. For days 1, 4, and 8, we performed one‐way ANOVA on the averages of biological replicates, followed by Dunnett´s Multiple Comparison test to detect significant differences between the mutants and the wild‐type. For day 14, we performed *t* test (** *p* < .01). (g) Quantification of amyloids in the wild‐type strain, *daf‐2(e1370),* and *daf‐16(mu86)*. Plots show mean (±*SD*) of four biological replicates. Statistics were performed as in (g) (* *p* < .05 and *** *p* < .001). (h) Recovery and development for larvae arrested during eight days in the presence of ammonium chloride and acridine orange (AO). (i) Recovery and development of wild‐type and *daf‐16* mutant L1 arrested during four days in the presence of (AO)

One possible explanation for the delayed reactivation of cell divisions is that the arrested larvae need to address the damage caused during prolonged arrest. Since quiescent L1 develop age‐related phenotypes (Roux et al., 2016), we investigated the process of L1 aging and whether it influences reactivation of the postembryonic development. Insulin/IGF‐1‐like signaling (IIS) is a prominent pathway in the control of aging in the adult (Kenyon, 2010) and a modulator of the survival of arrested L1 animals (Muñoz & Riddle, 2003). Reduced IIS leads to activation of DAF‐16/FOXO as it favors its translocation to the nucleus (Lin, Dorman, Rodan, & Kenyon, 1997; Ogg et al., 1997; Lin et al., 2001; Henderson & Johnson, 2001). Mutations in the sole *C. elegans* insulin receptor *daf‐2* increase survival during L1 arrest, while mutations in *daf‐16* reduce it (Baugh & Sternberg, 2006; Muñoz & Riddle, 2003). We measured recovery time in *daf‐16(mu86)*, *daf‐2(e1370),* and *daf‐16(mu86)*;*daf‐2(e1370)* double mutant. After one day of starvation, the recovery time of the *daf‐16* mutant was similar to that of the *wild‐type* strain. However, after four days of starvation, the *daf‐16* mutant showed a remarkable delay in recovery. For some animals, recovery took more than 50 hr (Figure 3a, left). Despite the long recovery time, the developmental timing of *daf‐16* is only mildly delayed compared to wild‐type (Figure 3a, right). The differential effect of the duration of quiescence on recovery and development is evident when calculating the fold change of the duration of both processes (Figure S3 a). This result suggests that DAF‐16 is necessary to maintain the potential to reactivate cell proliferation.

We then investigated whether increased DAF‐16 activation, as that found in *daf‐2(e1370)* mutants, leads to enhanced reactivation of proliferation. During prolonged L1 arrest, DAF‐16 relocalizes to the cytoplasm (Weinkove, Halstead, Gems, & Divecha, 2006), but *daf‐2* mutants show prolonged activation of DAF‐16 (Figure S3 b). When we measured recovery after one and four days of arrest, we did not observe a faster recovery of the *daf‐2* mutant (Figure S3 c). However, when we maintained L1 larvae in starvation for 14 days, the *daf‐2* animals recovered more rapidly than the wild‐type (Figure 3b left and Figure S3 d). This result is remarkable since low insulin signaling is generally related to proliferative defects (Michaelson, Korta, Capua, & Hubbard, 2010). The developmental time (M1‐M4) of *daf‐2* mutants is increased compared to wild‐type animals (Figure 3b, right), as previously described (Olmedo et al., 2015; Ruaud, Katic, & Bessereau, 2011). Importantly, the duration of arrest did not have a strong impact on growth rate, neither in wild‐type nor in *daf‐2* mutants (Figure 3b, right). As expected, *daf‐16* is epistatic to *daf‐2* for recovery from L1 arrest (Figure 3c), suggesting that the protective function of low insulin signaling requires DAF‐16 activity.

The delayed recovery of *daf‐16* mutants seems surprising, in light of the role of DAF‐16 as a repressor of proliferation (Baugh & Sternberg, 2006; Kaplan et al., 2015). We investigated whether the delayed recovery of *daf‐16* was caused by a defect to resume proliferation of blast cells by following seam cell division during recovery. After one day of arrest, seam cell divisions occurred at the same rate in the wild‐type and the *daf‐16* mutant. After four days of arrest, the delay in seam cell proliferation was much larger in the *daf‐16* mutant (Figure 3d). Even after 45 hr of recovery, many *daf‐16* animals continued arrested, with complete absence of blast cell divisions (Figure S3 e). After seam cell division, the time to M‐cell division was unaltered in the *daf‐16* mutant, even after four days of arrest (Figure S3 f).

We then investigated how activation of the transcription factor DAF‐16 enhances recovery. In the absence of food, DAF‐16 mediates both quiescence of blast cells through activation of the cyclin‐dependent kinase inhibitor CKI‐1 and the activation of stress resistance and maintenance pathways (Baugh, 2013; Baugh & Sternberg, 2006). Interestingly, the effect of DAF‐16 in cell quiescence has been separated from its effect in L1 survival. DBL‐1, a TGF‐β ligand, is necessary for reactivation of divisions during arrest in the *daf‐16* mutant. A double‐mutant *daf‐16;dbl‐1*, which does not undergo cell division during arrest, has the same L1 survival as the *daf‐16* mutant (Kaplan et al., 2015). This indicates that *daf‐16* mortality during arrest is not a consequence of inappropriate divisions. We looked at recovery of the double‐mutant *daf‐16;dbl‐1* and observed a delay in recovery greater than that of the single *daf‐16* mutant (Figure S3 g,h), indicating that *dbl‐1* is not epistatic to *daf‐16* for recovery from arrest. This result already suggests that, as it happens with survival, the delayed recovery of *daf‐16* is not a consequence of inappropriate cell divisions. When we analyzed *cki‐1* activation in the *daf‐16* mutant, we could not observe differences with the wild‐type strain (Figure S3 i). These results point to a role of DAF‐16 in cellular maintenance during L1 arrest that is independent of its role in the control of cell proliferation.

### 2.4 Markers of aging correlate with increased recovery time

In light of the previous results, we asked whether recovery time, as a proxy for reactivation of quiescent cells, correlated with the process of L1 aging. This would support the idea that recovery time reflects the level of activation of stress resistance and maintenance pathways during arrest. We investigated if recovery time was related to the accumulation of ROS, one of the markers of L1 aging (Roux et al., 2016). We stained L1 arrested larvae with Dihydroethidium (DHE), a compound that fluoresces in the presence of ROS. We observed an increasing variability in DHE signal over time of arrest (Figure S4 a). After 8 days of L1 arrest, we could visually establish two categories of larvae with high and low fluorescence in the head, which yielded significant differences when we quantified the actual fluorescent signal (Figure S4 b). We selected animals based on these two categories and analyzed their recovery, demonstrating that animals with higher DHE signals recovered significantly more slowly than those with lower signals (Figure 3e, left) but did not show developmental delay (Figure 3e, right). This result suggests that the capacity to recover depends on the accumulation of age‐related phenotypes (Roux et al., 2016).

We suspected that insulin signaling modulates the rate of accumulation of aging markers during L1 quiescence. The increase in the DHE signal was less pronounced in the *daf‐2* mutant, showing a significant reduction relative to the wild‐type animals after 14 days of arrest (Figure 3f). We monitored DHE signals in the *daf‐16* mutant up to day 8 of arrest, due to the increased mortality of these animals (Kaplan et al., 2015). At that time, the DHE signal was similar to that of wild‐type (Figure 3f), suggesting that either ROS accumulation does not account for the delay in reactivation of proliferation of this mutant or DHE staining does not reflect all ROS present in the nematode. We also checked DHE signal at day 11 of starvation and obtained similar results. At day 11, many *daf‐16* animals were dead, and they show very high DHE staining (Figure S4 c). However, this seemed to be a consequence, rather than the cause of death, since we do not observe a continuous transition in staining from live to dead L1s. The DHE signal in live worms is similar between wild‐type and *daf‐16* mutants (Figure S4 c). Next, we checked whether the delayed recovery of *daf‐16* mutants is due to a pronounced accumulation of other markers of aging. We analyzed the formation of amyloids using the dye NIAD‐4 (Habchi et al., 2016). In this case, the differences in staining in the *daf‐2* mutant after 14 days of arrest are even more pronounced. The mutant *daf‐16* showed significant differences in NIAD‐4 staining after 8 days of arrest (Figure 3g), suggesting that increased protein aggregation could delay recovery.

Another possible connection between DAF‐16 function and L1 recovery is lysosomal function. DAF‐16 promotes lysosome acidification in *C. elegans* (Baxi, Ghavidel, Waddell, Harkness, & Carvalho, 2017). Furthermore, impaired lysosomal function leads to delayed proliferation in rat embryonic fibroblast (Fujimaki et al., 2019). We tested whether lysosomal function had an effect on recovery time by applying compounds that alkalize lysosomes, namely ammonium chloride (NH_4_Cl) and acridine orange (AO) (Artal‐Sanz, Samara, Syntichaki, & Tavernarakis, 2006). After 8 days of treatment, L1 that were arrested in the presence of acridine orange (AO) showed a slower recovery than control animals. The treatment did not affect the subsequent developmental timing (Figure 3h and Figure S4 d). This experiment is of special relevance, as animals are exposed to AO only during L1 arrest. This result implies that recovery time can reflect the conditions during L1 arrest, independently of the conditions during the actual recovery period, which in this case are the same. When we analyzed the effect of AO in the *daf‐16* mutant arrested for four days, we observed an additional delay in recovery (Figure 3i), indicating that lysosomal function has a wider role in recovery from starvation.

Altogether, these results suggest that the physiological decline, similar to the aging process, that takes place specifically during L1 arrest leads to slower recovery once animals are fed.

### 2.5 Reduced maternal provisioning delays recovery in the progeny

*daf‐2* mutants produce longer, better‐provisioned embryos which, after prolonged starvation, produce more progeny than wild‐type (Hibshman, Hung, & Baugh, 2016). Reduced insulin signaling in this mutant increases maternal yolk provisioning to embryos and mitigates the reproductive abnormalities consequence of L1 starvation (Jordan et al., 2019).

**Figure 4:**
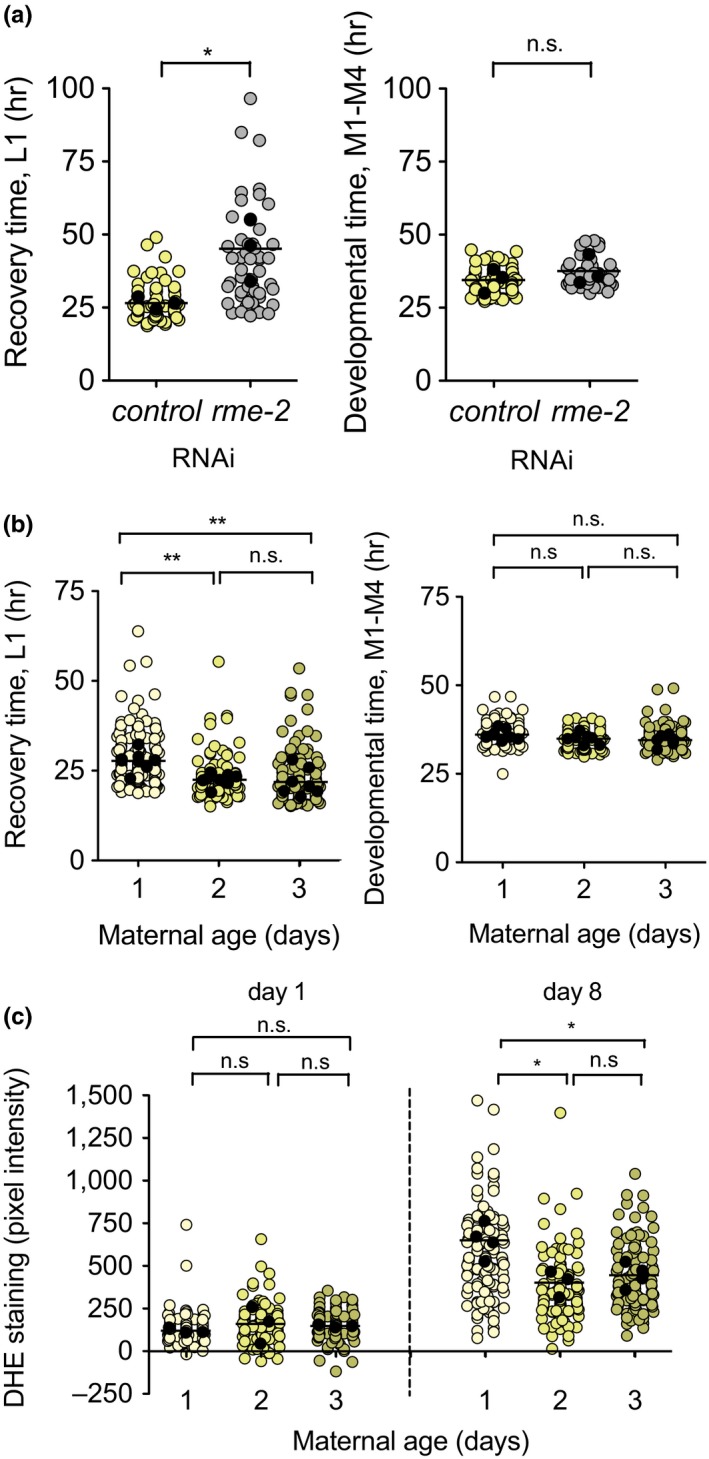
Maternal provisioning modulates recovery from L1 arrest. (a) Recovery time after 8 days of arrest for L1 larvae from mothers fed either control or *rme‐2* RNAi bacteria. We performed one‐way ANOVA followed by Bonferroni testing on the averages of 3 biological replicates (* *p* < .05). (b) Recovery and developmental time after 8 days of arrest of L1 larvae from day 1–3 progeny. We performed one‐way ANOVA followed by Bonferroni testing on the averages of 8 biological replicates (** *p* < .01). (c) ROS accumulation after 1 or 8 days of arrest for day 1–3 progeny. We performed one‐way ANOVA followed by Bonferroni testing on the averages of 3–4 biological replicates (* *p* < .05)

We investigated whether modulation of maternal provisioning per se affected recovery of the progeny. Vitellogenin/yolk proteins uptake by the oocyte occurs through RME‐2 mediated endocytosis (Grant & Hirsh, 1999). Although yolk is not necessary for efficient reproduction in *C. elegans* (Van Rompay et al., 2015), efficient yolk protein transport and storage contribute to L1 survival (Chotard, Skorobogata, Sylvain, Shrivastava, & Rocheleau, 2010). We tested the efficiency of the *rme‐2* knockdown by measuring VIT‐2::GFP in embryos of animals treated with *rme‐2* RNAi (Figure S5 a,b). We obtained L1 larvae after treatment of the mothers with *rme‐2* RNAi and with control bacteria and maintained them in arrest for 8 days. Larvae from embryos with reduced vitellogenin showed longer recovery times than larvae from mothers treated with control RNAi (Figure 4a, left). Despite the pronounced delay in recovery, developmental timing was similar between conditions (Figure 4a, right). Our results, therefore, confirm that yolk provisioning is important during L1 starvation and provides a mechanistic link between insulin signaling and recovery from L1 arrest.

Interestingly, differential embryo provisioning of vitellogenin or yolk proteins, as a consequence of maternal age is one factor introducing phenotypic variability in development after extended arrest. Embryos from older mothers contain more yolk proteins and develop faster (Perez, Francesconi, Hidalgo‐Carcedo, & Lehner, 2017). A recurrent observation for both recovery time and aging markers is an increase in the coefficient of variation as the values increase. This means that processes that increase recovery time show an important interindividual variability. The causes of such variability in genetically identical organisms reared in the same environmental conditions are only starting to be elucidated. We investigated whether naturally occurring differences in maternal provisioning could explain the variability we observed in recovery time and aging markers. We obtained embryos from gravid adults in their first, second, and third day of egg laying, maintained them in L1 arrest for 8 days, and measured its recovery time. We found that larvae from older mothers recovered significantly faster than those of younger mothers (Figure 4b). Although larvae from day 3 mothers were on average similar to those from day 2 mothers, this population contained the fastest animals in terms of recovery. When analyzing the 50% fastest animals in each condition, average recovery was progressively faster, with recovery times of 22.52, 19.65, and 17.56 hr respectively (Figure S5 c). This effect was more prominent when we analyzed recovery of the very first embryos produced by the mothers (day 0.5, Figure S5 d). Maternal age does not have an effect on developmental timing after extended arrest (Figure 4c and Figure S5 d).

We also analyzed whether embryos from older mothers were protected against the accumulation of L1 aging markers. After 8 days of arrest, larvae from day 2–3 mothers showed reduced DHE accumulation compared to larvae from day 1 mothers (Figure 4c). We found no differences in NIAD‐4 staining (Figure S5 e). These results support a role for maternal age in preventing L1 aging, leading to maintenance of proliferation potential. To explore a possible link between insulin signaling and yolk provisioning, we analyzed the localization of DAF‐16 during L1 arrest, in larvae treated with *rme‐2* RNAi (Figure S5 f). Larvae with reduced yolk provisioning had similar levels of nuclear DAF‐16 than those treated with control RNAi. This result indicates that reduced L1 aging of larvae from older mothers is unlikely to be due to an increased activation of DAF‐16.

## 3 DISCUSSION

The balance between cell quiescence and proliferation is crucial for maintenance of stem cell pools, which hold the potential to maintain tissue homeostasis or replace dead cells after injury. On the one hand, stimulation of proliferation of quiescent cells provokes the exhaustion of stem cells (Li & Clevers, 2010). On the other, the degenerative processes in stem cells and the systemic cues that regulate their activity have been connected to the age‐dependent decline in regenerative potential of tissues (Ahmed, Sheng, Wasnik, Baylink, & Lau, 2017). Proliferative potential of stem cells is reduced over time in quiescence. Now, we have shown that, also in *C. elegans*, prolonged arrest delays blast cell divisions. This finding parallels that in cultured cells, where delayed reactivation of divisions after prolonged starvation is proposed to be due to quiescence deepening (Kwon et al., 2017). This suggests that blast cells in L1 larvae progress to deeper levels of quiescence throughout L1 arrest. In *C. elegans*, we have monitored this process over 27 days of arrest, showing that the selective effect of prolonged quiescence on recovery extends over the survival of arrested L1. These results support the relevance of L1 arrest upon starvation as a model to study proliferative potential after quiescence in vivo.

Roux et al. proposed that quiescent L1 suffer a physiological decline similar to the aging of the adult (Roux et al., 2016). Most aging markers accumulated by L1 larvae during arrest, including ROS, were reversed by feeding, and the competence of the animal to clear those signs of aging determined the capacity to recover from L1 arrest. Here, we show that we can measure the proliferative potential of quiescent L1, not only in terms of recovery per se, but through precise quantitation of recovery time. Through determination of the recovery time of *daf‐2* mutants, we found that low insulin signaling plays a maintenance role during quiescence, as *daf‐2* mutants recover faster than the wild‐type after prolonged arrest. This finding seems surprising considering the role of this pathway in proliferation. However, we have observed reduced ROS accumulation and protein aggregation in the *daf‐2* mutant. Aged stem cells accumulate reactive oxygen species (ROS), DNA damage, aggregated proteins, and they show mitochondrial misfunction (reviewed in (Oh, Lee, & Wagers, 2014)). Interestingly, a recent work has determined that lysosomal gene expression increases as quiescence deepens. Lysosomal function favors a shallower level of quiescence by reducing ROS accumulation. A score for gene expression during the deepening of quiescence parallels cellular senescence and aging (Fujimaki et al., 2019). This recent finding and our own results suggest that ROS accumulation favors deeper quiescence. We have shown that lysosome malfunction also increases recovery time after prolonged quiescence in *C. elegans,* which represents an important parallelism to the recent findings in rat embryonic fibroblasts (Fujimaki et al., 2019). Since DAF‐16 drives lysosomal function in *C. elegans* (Baxi et al., 2017), this could explain the observation that *daf‐2(e1370)* mutants*,* which present a constitutive DAF‐16 activation, show lower levels of ROS and faster recovery. Although FoxO deficient hematopoietic stem cells present increased ROS (Tothova et al., 2007), we do not observed higher ROS accumulation in a *daf‐16* mutant. It is possible that other factors, as protein aggregation, are limiting survival of this mutant before the increased accumulation of ROS becomes evident. Our results indicate that activation of DAF‐16 confers an advantage for recovery, possibly by reducing the rate of L1 aging. Despite the proliferative role of insulin signaling, its repression could be beneficial for maintenance of cells by conferring a shallower level of quiescence, allowing faster recovery upon feeding. It is also important to notice that insulin signaling has already been connected to the progression of L1 aging through the regulation of sleep. However, *daf‐16* mutation alone does not impair L1 sleep (Wu et al., 2018), and therefore, sleep loss could not explain the defective recovery of this mutant.

The delayed L1 recovery after prolonged starvation involves a delay in reactivation of blast cell divisions. This process is, in principle, controlled by insulin signaling, as *daf‐16* mutants are arrest defective. However, it is important to recall that the defective‐arrest phenotype of *daf‐16* requires the presence of ethanol (Fukuyama et al., 2015). In our experiments, we do not include ethanol in the buffer used for the starvation assay, indicating that the phenotypes we observe are not related to reactivation of cell divisions during arrest. Actually, we have found that *daf‐16* mutants show activation of the cell cycle inhibitor *cki‐1*/CIP/KIP/p27, unlike what is observed when ethanol is present in the starvation media (Baugh & Sternberg, 2006). This means that, as previously suggested by other authors, additional pathways could contribute to the regulation of *cki‐1*/CIP/KIP/p27 during L1 arrest (Baugh, 2013; Kniazeva, Euler, & Han, 2008). Interestingly, protein aggregates usually sequester proteins involved in cell cycle control (Lutz & Peng, 2018; Zhou et al., 2004). The increased protein aggregation that we observed in *daf‐16* could lead to a reduction of cell cycle regulators and delay its progression.

One feature of *daf‐2* animals is that they produced longer, better‐provisioned embryos than the wild‐type strain. We modulated maternal provisioning by performing RNAi against the vitellogenin/yolk transporter *rme‐2* and by controlling maternal age of the L1 larvae subjected to arrest. These interventions were sufficient to modulate L1 aging and affect recovery time. This observation provides a possible mechanistic link between reduced insulin signaling and the regulation of L1 aging. The effect of maternal age in ROS accumulation and in recovery also explains our observation of increased variability upon prolonged starvation in the experiments where we did not control for maternal age. The heterogeneity in recovery of larvae with different maternal provisioning is reminiscent of the effect of cell growth and divisions history in subsequent quiescence exit in rat embryonic fibroblasts (Wang et al., 2017). Although we have not tested this relationship specifically, it is tempting to speculate that maternal dietary conditions and growth rate will affect recovery of the progeny.

## 4 EXPERIMENTAL PROCEDURES

### 4.1 Culture conditions and strains

We cultured stock animals according to standard methods (Brenner, 1974), maintaining them at 20°C on nematode growth medium (NGM) with a lawn of *Escherichia coli* OP50‐1. The only exception is in Figure 1a–d, where animals were maintained at 18°C previous to the experiment. A detailed description of the strains used in this work is presented in the Supporting information.

### 4.2 Preparation of starved L1 and recovery

For starvation experiments, we first treated gravid adults with alkaline hypochlorite solution to obtain embryos. For all experiments, we adjusted the concentration to 20 embryos/µl of M9 buffer. The embryos were incubated at 20°C, with gentle shaking, leading to hatching, and arrest at the L1 stage. After the corresponding amounts of time, arrested L1 were used for the different experiments. For recovery experiments, we staggered the hypochlorite treatments to be able to analyze, simultaneously, animals arrested for different periods of time. For the analysis of ROS and protein aggregation during arrest, we followed the same cohort over the relevant periods of time, usually monitoring at day 1, 4, 8, and 14 of L1 arrest.

For recovery experiments, we resumed development by adding one volume of 20 g/L *E. coli* OP50‐1 in S‐basal (with cholesterol) to the arrested L1, to have a final concentration of 10 g/L *E. coli* OP50‐1.

### 4.3 Luminometry of single worms

We measured recovery time and developmental timing using a bioluminescence‐based method (Olmedo et al., 2015). Briefly, L1 arrested animals were placed individually in wells of a white 96‐well plate containing 100 µl of S‐basal (including 5 µg/ml cholesterol) with 200 µm Luciferin. After all animals were placed in the wells, we added 100 µl of S‐basal containing 20 g/L *E. coli* OP50‐1 per well to resume development simultaneously for all the animals. We used sample sizes >20 individually quantified animals in at least three biological replicates. We alternated the samples across the plate to avoid local effects (i.e., temperature of the reader).

### 4.4 Analysis of seam and M‐cell divisions

For the experiments with the single reporters, we obtained about 300 µl of L1 larvae suspension of the strains GAL69, JR667 (seam cell reporters), or PD4666 (M‐cell reporter) arrested for one day or four days. On the day of the experiment, we added bacteria to all samples simultaneously to initiate recovery. At the indicated times, we collected 30 µl to a clean 1.5 ml tube and centrifuged for 1 min at 850 *g*, in a tabletop centrifuge. We removed 25 µl of supernatant and added 1 µl of 100 mm Levamisole. After the addition of food, cell divisions were monitored every hour over 3–14.5 hr (V seam cells) or 6.5–20.5 hr (H seam cells and M cells). Sampling times were displaced in time among independent experiments to maximize the information to be obtained from the time course. We calculated the percentage of animals with divisions over a population of at least 40 animals per time point and condition.

For the experiments with the double‐seam and M‐cell reporter, we prepared embryos twice per condition, separated by 12 hr. This way we could resume development of these animals with a 12‐hr difference, allowing monitor divisions in the first 24 hr over an experimental period of 12 hr. We obtained L1 larvae of the strains MOL198 and MOL253 arrested for one day or four days and then resumed development by the addition of food, as indicated above. We calculated the percentage of animals with divisions over a population from at least 40 animals per time point and condition. In order to calculate the time at which 50% of the developing population had divisions, we fitted the data to a cumulative Gaussian distribution and calculated the mean value. In both cases, we monitored the animals using a Leica scope DMi8 using GFP excitation/emission filters.

### 4.5 Subcellular localization of DAF‐16

We obtained L1 larvae of the strain TJ356 arrested for one day or four days and initiated the recovery by adding food. At the indicated times, we took an 18 µl aliquot of the suspension and placed it on a microscope slide with 2 µl of 10 mm Levamisole. We used a reduced concentration of Levamisole in order to avoid artificial translocation of DAF‐16. We visualized the localization of DAF‐16 in a Leica scope DMi8 using GFP excitation/emission filters and counted the animals with nuclear, cytoplasmic, or intermediate localization. Since the localization of DAF‐16 can be affected by temperature, the temperature of the room was maintained at 20°C during visualization of the animals. We categorized animals as having nuclear, intermediate, or cytoplasmic localization, in a population of at least 100 larvae. Since this categorization is somehow subjective, the experimenter was blind to the condition tested.

### 4.6 Analysis of *cki‐1* activation

We obtained L1 larvae of the strain VT825 and MOL270 arrested for one day or four days. When relevant, we resumed development by addition of food. At the indicated times, we transferred 50 µl of the suspension to a clean tube and centrifuged 1 min at 850 *g*. We removed 50 µl of the supernatant and added 1 µl of 100 mm Levamisole. We transferred the sample to a glass slide and cover and visualized the GFP signal in a Leica scope DMi8 using GFP excitation/emission filters. We counted the fraction of animals with GFP signal in the seam cells (categorized as induced *cki‐1*), in a population of at least 50 larvae.

### 4.7 DHE staining and longitudinal analysis of recovery

We added Dihydroethidium (DHE, Sigma) at a final concentration of 10 µm in 30 µl of arrested L1. After incubation for 2 hr at 20°C, we centrifuged the sample, removed 25 µl of supernatant, and added 1 µl of 100 mm Levamisole. We placed 5 µl of the anesthetized L1´s on a microscope slide with a cover slip. We imaged the animals in a Leica scope DMi8 at 200x magnification using transmitted light and *mCherry* excitation/emission filters. For quantification, we drew ROIs around the head of the larvae using the image from transmitted light and then quantified pixel intensity in the red channel using Image J. We subtracted the background for each image. We measured DHE of at least 15 animals per conditions in 3–4 biological replicates.

For the longitudinal analysis of recovery, we stained L1 larvae arrested for 8 days with DHE, as above, and then categorized them into animals with low or high signal. We collect animals from both categories to measure recovery and developmental timing as above.

### 4.8 NIAD‐4

We added NIAD‐4 (Cayman Chemicals) at a final concentration of 1 µm (0.1% DMSO in M9 buffer) to an aliquot of 30 µl of arrested L1. After incubation for 2 hr at 20°C, we centrifuged the sample, removed 25 µl of supernatant, and washed with 25 µl of M9 buffer to remove the excess NIAD‐4. After centrifugation, we removed again 25 µl and added 1 µl of 100 mm Levamisole. We placed 5 µl of the anesthetized L1 on a microscope slide for analysis. We imaged the animals in a Leica scope DMi8 at 200× magnification using transmitted light and *mCherry* excitation/emission filters. Quantification was performed as for DHE staining. We measured NIAD‐4 of at least 15 animals per conditions in four biological replicates.

### 4.9 NH_4_Cl and acridine orange treatment

After hypochlorite treatment to obtain embryos, we incubated them in M9 containing 5 mm ammonium chloride (NH_4_Cl) or 100 mm acridine orange (AO) for eight days. These chemicals were diluted >10^4^ times during the preparation of the plate for the luminometer, to avoid possible effects of the compounds on larval development.

### 4.10 Synchronization of mothers

To obtain embryos from mothers in their first, second, and third day of egg laying, we prepared synchronized populations allowing 20 gravid adults to lay eggs on NGM plates for two hours. After this period, we removed the gravid adults and allowed the progeny to grow at 20°C. After approximately 80, 88, 104, 112, 128, and 136 hr, the progeny were, respectively, at what we have named day 0.5, 1, 1.5, 2, 2.5, and 3 of egg laying. Day 0.5 refers to the time when the first embryos have been produced, while at day 1 all animals (initially synchronized by 2 hr) have started egg laying.

### 4.11 Yolk reduction by *rme‐2* RNAi

We obtained embryos with reduced yolk by alkaline hypochlorite treatment of gravid adults grown on *rme‐2* RNAi from the Ahringer library. We cultivated *rme‐2* RNAi bacteria overnight and diluted 1/10 with control bacteria HT115 containing the empty plasmid pL4440. We added 500 µl of the diluted RNAi or control bacteria on NGM plates with 1 mm IPTG and 100 μg/ml ampicillin. We let the bacterial lawn dry and incubated the plates for 5 hr at 37°C and overnight at room temperature. We transferred 20 gravid adults of the strain MRS387 per plate and let them lay eggs for 2 hr before retiring them from the plates. We grew them for 4 days at 20°C before proceeding with the hypochlorite treatment. We maintained the animals in L1 arrest for 8 days at 20°C. Development was re‐initiated by adding food, and we monitored development as previously described. As a control for the RNAi treatment, we used the strain RT130 to monitor VIT‐2::GFP transport into oocytes.

### 4.12 Data analysis and statistics

We analyzed luminometry data as previously described (Olmedo et al., 2015)**.** For all luminometry experiments, we have plotted the values for each individual animal and also the average of the values for each biological replicate. For statistics, we have used the averages of independent biological replicates to avoid the inflated *N* value from using individual animals. The averages of most groups were normally distributed but in cases when N was too low to assess normality, we visually inspected the values to discard important deviations from normality and variances were similar. We used unpaired two‐tailed *t* test to compare the means of two groups and one‐way ANOVA to compare more than two groups. ANOVA was followed by Bonferroni´s post hoc to compare all groups or by Dunnett´s test to compare groups to a control condition (mutants vs. wild‐type). For the analysis of DAF‐16 localization in wild‐type and *daf‐2* mutants over time in arrest, we have performed Two‐way ANOVA.

## CONFLICT OF INTEREST

The authors declare no competing financial interests.

## AUTHOR CONTRIBUTIONS

M.O., A.M‐C., M.J.R‐P., S. G‐S., and A. F‐Y. carried out the experiments; M.O. and A.M‐C. designed the experiments; M.O., A.M‐C., M.J.R‐P., M.M., and M.A‐S. interpreted results; and M.O., A.M‐C., M.M., and M.A‐S. wrote the manuscript.

## Data Availability

The data that support the findings of this study are openly available in Mendeley Data at http://doi.org/10.17632/srwvfgf42p.1.
