# Supplementary material for: Prolonged quiescence delays somatic stem cell‐like divisions in Caenorhabditis elegans and is controlled by insulin signaling
Source: Aging Cell. 2019 Dec 18;19(2):e13085. doi: 10.1111/acel.13085 (PMC6996950; doi:10.1111/acel.13085)
Supplement: Supplementary file 1 [file ACEL-19-e13085-s001.pdf]

Supporting information

Prolonged quiescence delays somatic stem cell-like divisions in *Caenorhabditis elegans* and is controlled by insulin signaling

María Olmedo[1,*], Alejandro Mata-Cabana[1], María Jesús Rodríguez-Palero[2,3], Sabas García-Sánchez[1], Antonio Fernández-Yañez[2,3], Martha Merrow[4] and Marta Artal-Sanz[2,3,*]

[1]Departamento de Genética, Facultad de Biología, Universidad de Sevilla, Avenida Reina Mercedes s/n, 41012 Seville, Spain

[2]Andalusian Center for Developmental Biology. Consejo Superior de Investigaciones Científicas/Junta de Andalucía/Universidad Pablo de Olavide. Carretera de Utrera Km 1, 41013 Seville, Spain.

[3]Department of Molecular Biology and Biochemical Engineering, Universidad Pablo de Olavide. Carretera de Utrera Km 1, 41013 Seville, Spain.

[4]Institute of Medical Psychology, Faculty of Medicine, LMU Munich, Goethestrasse 31, 80336 Munich, Germany.

Correspondence:

María Olmedo: mariaolmedo@us.es

Marta Artal-Sanz: martsan@upo.es

This file contains:

Supporting text: detailed description of strains

5 Supplementary figures

Strains

For the initial characterization of the recovery time and developmental timing in response to starvation (Fig. 1c-f), we used the reporter strain PE255 *feIs5* [*Psur-5::luc+::gfp; rol-6 (su1006)*]X (Lagido et al. 2008). For subsequent experiments we created a new luciferase reporter without the marker *rol-6*, generating the strain MRS387 *sevIs1 [Psur-5::luc+::gfp]X* and MRS389 *sevIs2 [Psur-5::luc+::gfp]*. To generate MRS387 and MRS389 we injected the plasmid pSLGCV in N2 worms and selected several lines that transmitted the array. We then irradiated one of these strains MRS222 *sevEx1[Psur-5::luc+::gfp]* with X-rays to integrate the reporter array. These strains were outcrossed 10 times with N2. We crossed the new reporter strains, using standard genetic techniques, to generate MRS424 *daf-16(mu86)I; sevIs1 [Psur-5::luc+::gfp]X*, MRS434 *daf-2(e1370)II; sevIs1 [Psur-5::luc+::gfp]X*, MOL56 *daf-2(e1370)III; daf-16(mu86)I; sevIs1[Psur-5::luc+::gfp]X*, MOL174 *dbl-1(wk70); sevIs1[Psur-5::luc+::gfp]X*, and MOL257 *dbl-1(wk70); daf-16(mu86); sevIs1[Psur-5::luc+::gfp]X.*

For the analysis of cell divisions in response to feeding we used the strains GAL69 *matIs38 [Pscm::CYB-1 DB-mCherry::unc-54 3' UTR; Pscm::NLS-GFP::tbb-2 3' UTR; Pmyo-2::GFP]*, PD4666 *ayIs6 [hlh-8::GFP fusion + dpy-20(+)],* JR667 *unc-119(e2498::Tc1)III; wIs51[SCMp::GFP + unc-119(+)]V* and the double seam and M cell reporters MOL198 *matIs38 [Pscm::CYB-1 DB-mCherry::unc-54 3' UTR; Pscm::NLS-GFP::tbb-2 3' UTR; Pmyo-2::GFP]*; *ayIs6 [hlh-8::GFP fusion + dpy-20(+)]* and MOL253 *daf-16(mu86) matIs38 ayIs6.* To assess *cki-1* activation we used the reporter strain VT825 *dpy-20(e1282);maIs113 [cki-1::GFP + dpy-20(+)],* that we later crossed with CF1038 *daf-16(mu86)* to generate MOL270. For the analysis of ROS and accumulation of amyloids, we used the strains N2, CB1370 *daf-2(e1370)*, and CF1038 *daf-16(mu86).* To assess the localization of DAF-16 we used TJ356 *zIs356[Pdaf-16::daf-16a/b-gfp; rol-6]* IV. As a control for yolk reduction by RNAi treatment, we used RT130 *unc-119(ed3); pwIs23 [Pvit-2::vit-2::gfp, unc119(+)].*

Figure S1

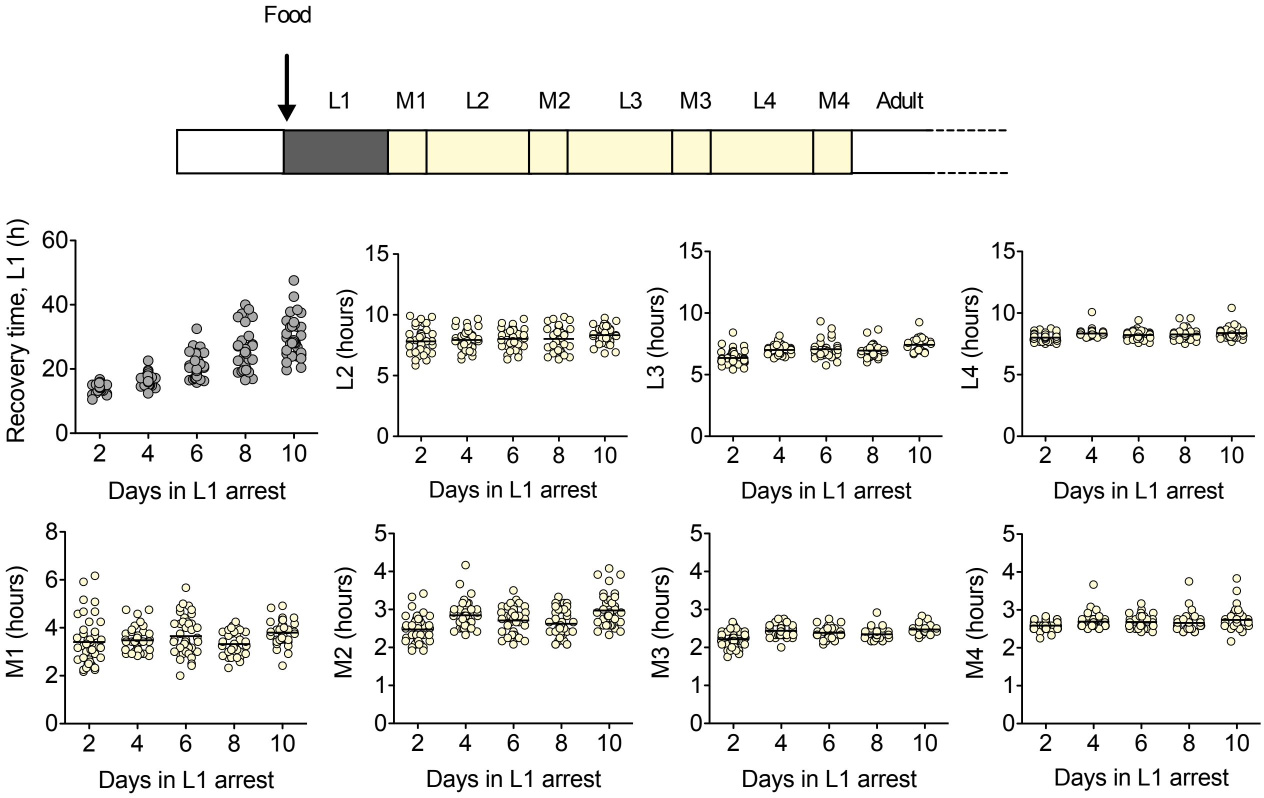

Figure S1. Relative to figure 1.

Duration of each stage of development upon feeding after 2-10 days of L1 arrest. Larval stages (L1-L4) and molts (M1-M4).

Figure S2

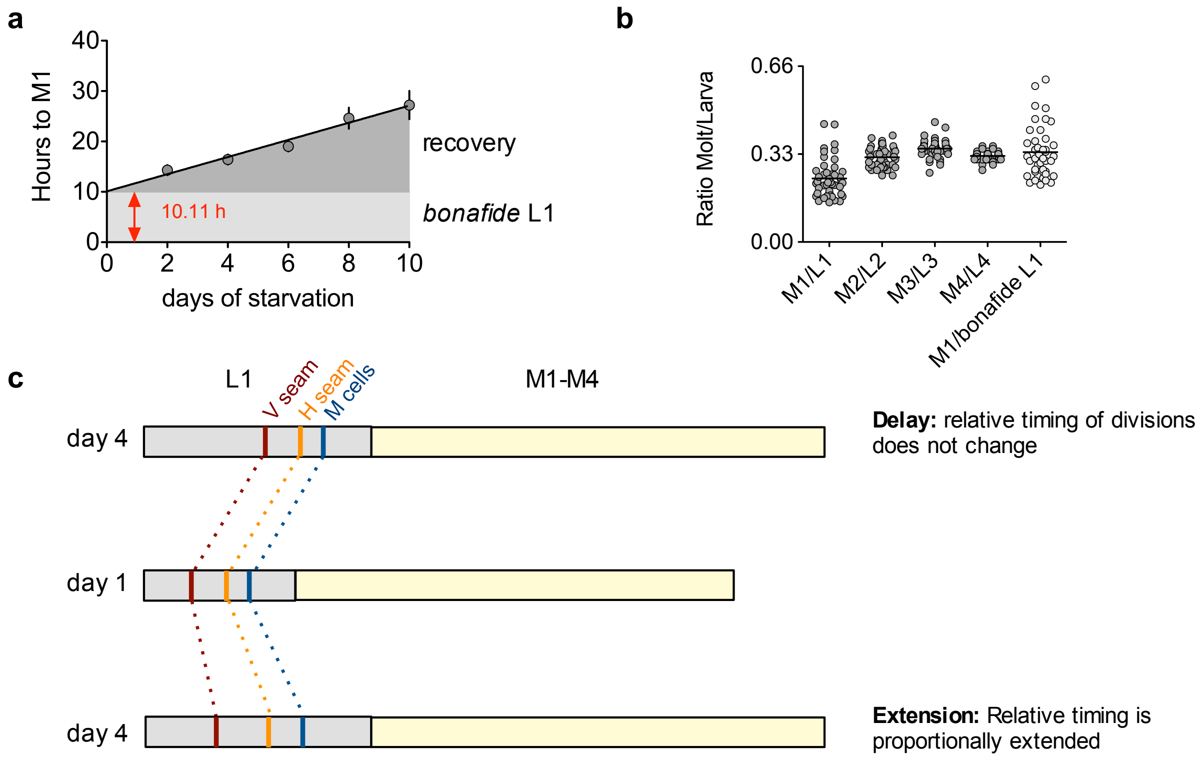

Figure S2. Relative to figure 2.

a. Linear regression of the average values in Fig. 1d and estimation of the *bonafide* L1 as the time necessary to enter the first molt from the point of arrest. **b.** Molt/larva ratios for all larval stages using the actual data after two days of arrest or using the calculated *bonafide* L1. **c.** Alternative explanations of the longer recovery time (L1) after prolonged quiescence. Our data fits with a delay in the initiation of postembryonic development.

Figure S3

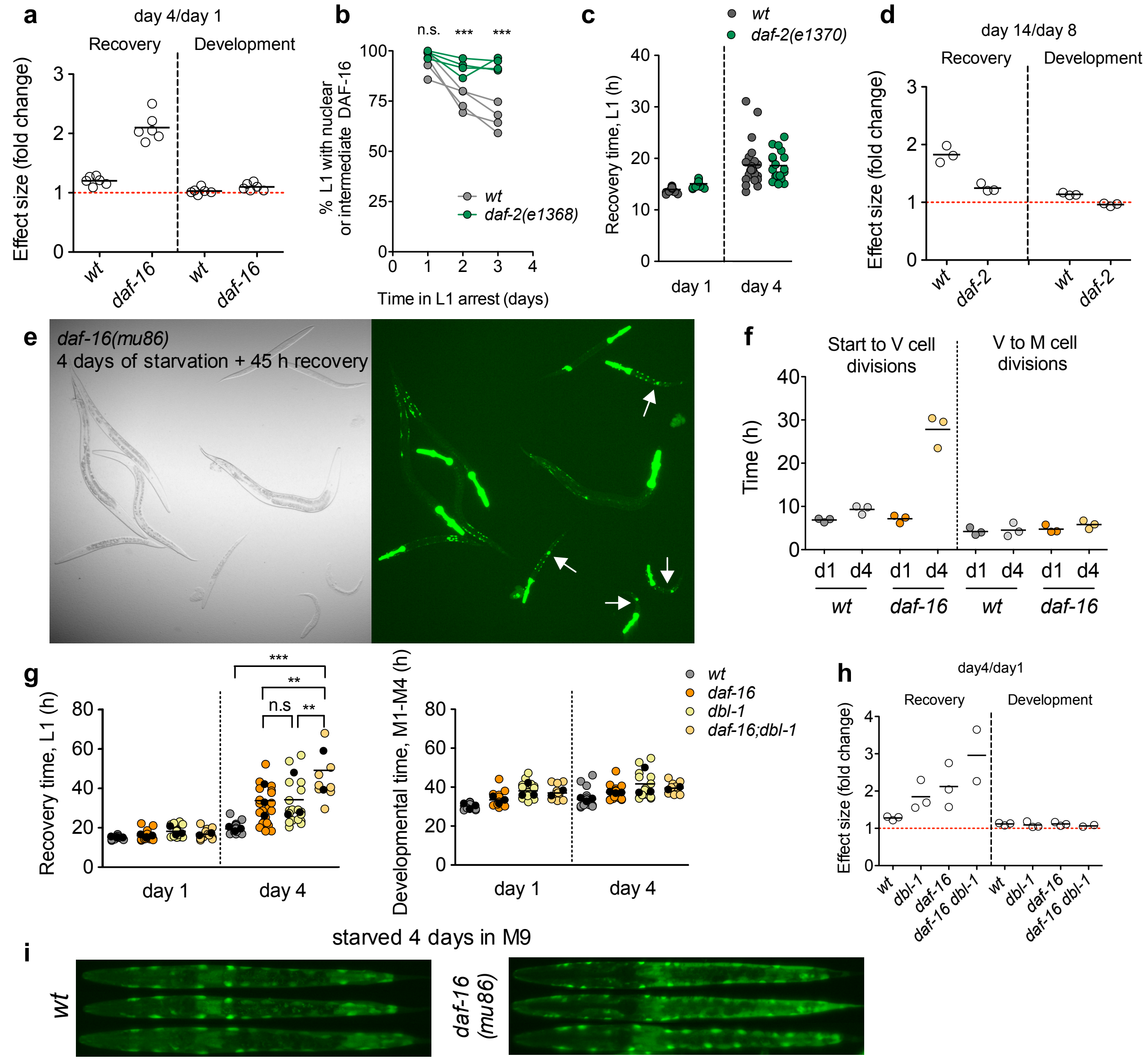

Figure S3. Relative to figure 3.

a. Effect size of prolonged arrest in the *daf-16* mutant. The plot shows the fold change in the six independent experiments, represented in Fig. 3a and Fig. 3c. **b.** Percentage of *wt* and *daf-2(e1368)* L1 larvae with nuclear or intermediate localization of DAF-16::GFP. We performed Two-way ANOVA followed by Bonferroni testing on the data from four biological replicates (*** $p<0.001$). **c**. Recovery for the wild-type strain and the *daf-2(e1370)* mutant after one and four days of arrest. **d.** Effect size of prolonged arrest in the *daf-2* mutant, for the three experiments shown in Fig. 3b. **e.** Representative image of *daf-16* mutants after 45 hours of recovery following four days in L1 arrest. Arrows point at animals with undivided seam and M cells. **f.** Relative timing of V seam and M cell divisions in *daf-16* mutants after one or four days of arrest. **g.** Recovery and developmental time for the wild-type strain, *daf-16*, *dbl-1* and the double mutant *daf-16 dbl-1* after one and four days of starvation. The plot shows data for 2-3 experiments. Statistics results from One-way ANOVA performed over all the replicates. ** $p<0.01$, *** $p>0.001$. **h**. Effect size of prolonged arrest in the mutants shown in g). **i**. Representative images of *cki-1* activation in wt and *daf-16(mu86)* after four days of L1 arrest.

Figure S4

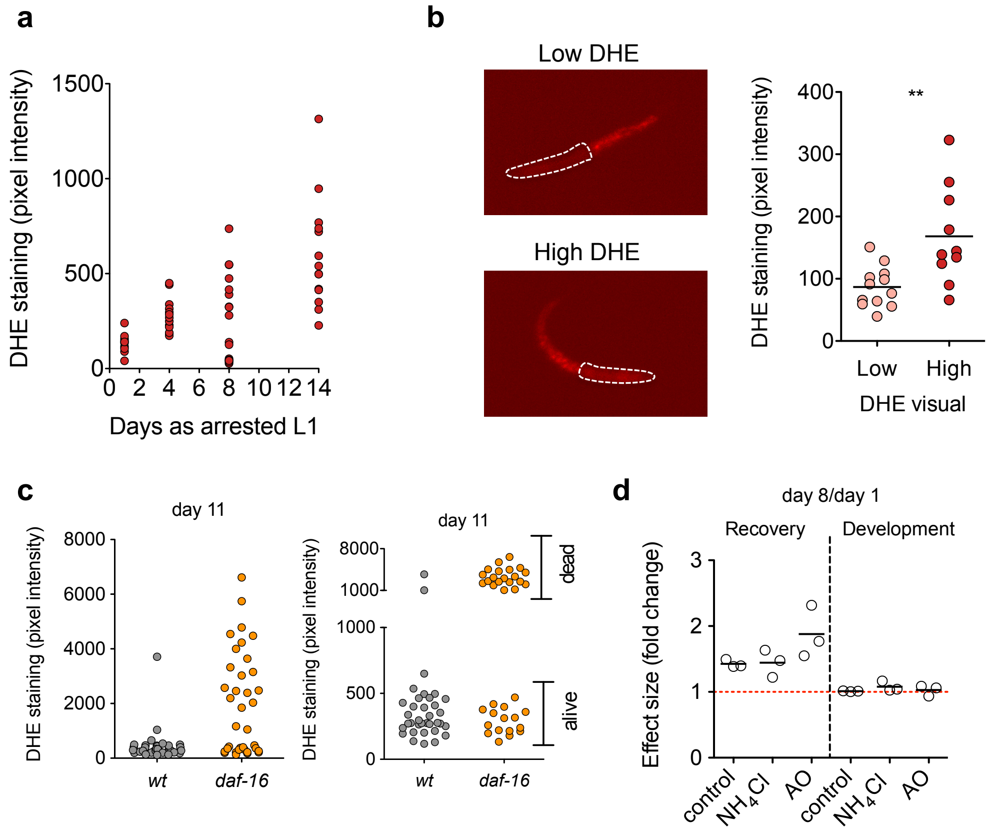

Figure S4. Relative to figure 3.

a. ROS accumulation over time in L1 arrest. The plot shows data from at least 10 animals per condition. **b.** Representative pictures of animals with low and high DHE signal after 8 days of starvation (left panel) and quantification of DHE staining in animals visually categorized as having low or high signal (right panel). **c.** Quantification of DHE staining in *daf-16* larvae starved for 11 days. The animals with signal >1000 (relative pixel intensity) corresponded to dead animals. **d.** Effect size of AO treatment in recovery and development.

Figure S5

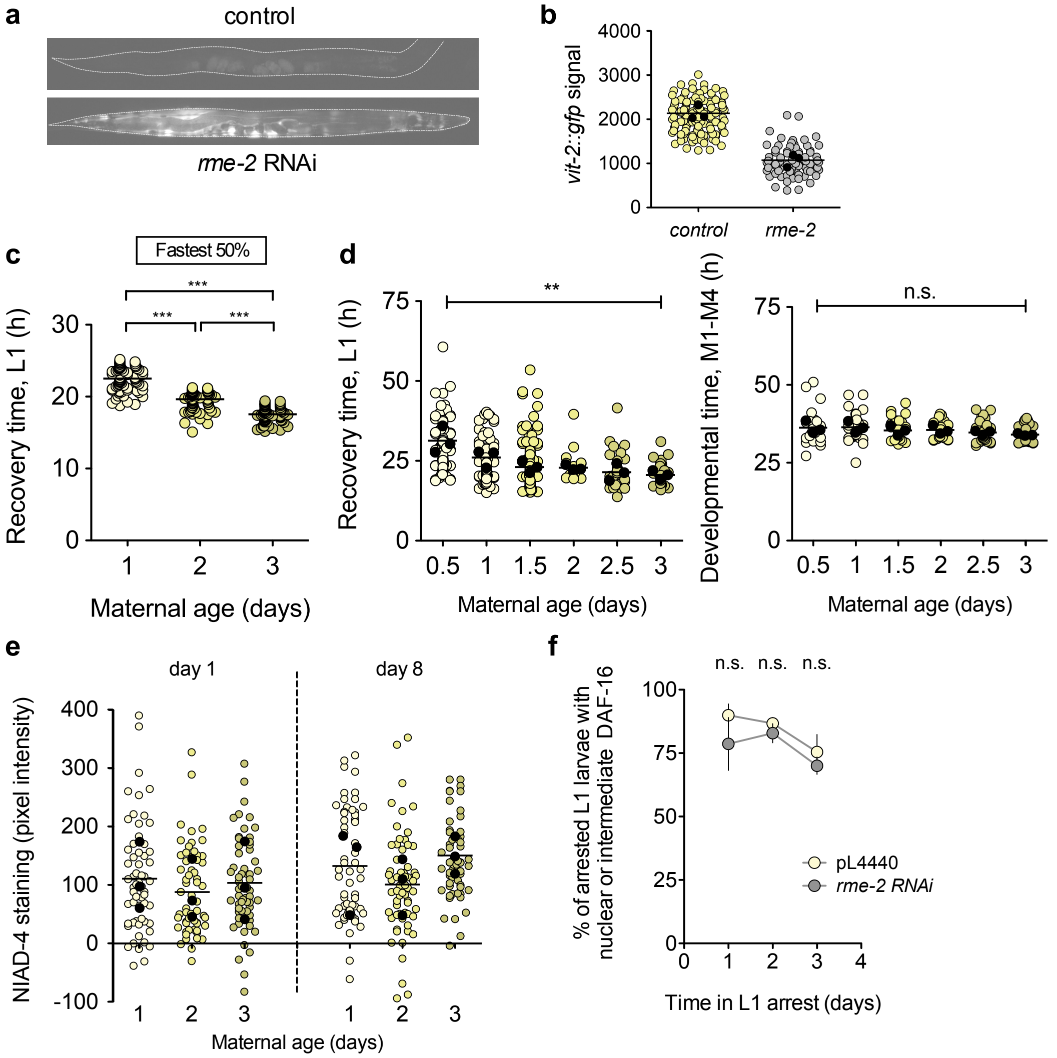

Figure S5. Relative to figure 4.

a. Representative pictures of *rme-2* RNAi treated and control worms used for assessment of the RNAi treatment. The pictures were acquired with the same intensity and exposure and are shown with the same level of brightness. **b.** Quantification of VIT-2::GFP in embryos of control and *rme-2* RNAi treated animals**. c.** Recovery time of the fastest half of the population shown in (Fig. 4b). We performed One-way ANOVA followed by Bonferroni testing on the values from individual animals (*** $p<0.001$). **d.** Recovery time and developmental timing after eight days of L1 arrest from progeny collected at the indicated maternal age (0.5-3 days of egg laying). Average values per experiment are indicated with a black dot, and values from single animals are indicated with colored dots. We performed One-way ANOVA followed by Bonferroni testing on the averages of 3 biological replicates (** $p<0.01$). **e.** NIAD-4 accumulation in day 1-3 progeny after one and eight days of arrest. The plot shows data from 3 biological replicates. Average values per experiment are indicated with a black dot, and values from single animals are indicated with colored dots. **f.** Percentage of L1 larvae with nuclear or intermediate localization of DAF-16::GFP, for animals treated with pL4440 or *rme-2* RNAi. We performed Two-way ANOVA followed by Bonferroni testing on the data from four biological replicates.
